# Pre-diagnostic body mass index and weight change in relation to colorectal cancer survival among incident cases from a population-based cohort study

**DOI:** 10.1186/s12885-016-2445-4

**Published:** 2016-07-07

**Authors:** Ida Laake, Inger K. Larsen, Randi Selmer, Inger Thune, Marit B. Veierød

**Affiliations:** Oslo Centre for Biostatistics and Epidemiology, Institute of Basic Medical Sciences, University of Oslo, Oslo, Norway; Department of Vaccines, Norwegian Institute of Public Health, Oslo, Norway; Department of Registration, Cancer Registry of Norway, Oslo, Norway; Department of Pharmaco-epidemiology, Norwegian Institute of Public Health, Oslo, Norway; Department of Community Medicine, University of Tromsø, Tromsø, Norway; Department of Oncology, Oslo University Hospital, Oslo, Norway; Department of Nutrition, Institute of Basic Medical Sciences, University of Oslo, Oslo, Norway

**Keywords:** Colorectal cancer, Survival, Body mass index, Weight change, Cohort study

## Abstract

**Background:**

Whether excess body weight influences colorectal cancer (CRC) survival is unclear. We studied pre-diagnostic body mass index (BMI) and weight change in relation to CRC-specific mortality among incident CRC cases within a large, Norwegian cohort.

**Methods:**

Participants’ weight was measured at health examinations up to three times between 1974 and 1988. CRC cases were identified through linkage with the Norwegian Cancer Registry. In total, 1336 men and 1180 women with a weight measurement >3 years prior to diagnosis were included in analyses. Hazard ratios (HRs) and confidence intervals (CIs) were estimated with Cox regression.

**Results:**

During a mean follow-up of 5.8 years, 507 men and 432 women died from CRC. Obesity (BMI ≥30 kg/m^2^) was associated with higher CRC-specific mortality than normal weight (BMI 18.5–25 kg/m^2^) in men with proximal colon cancer, HR = 1.85 (95 % CI 1.08–3.16) and in women with rectal cancer, HR = 1.93 (95 % CI 1.13–3.30). Weight gain was associated with higher CRC-specific mortality in women with CRC, colon cancer, and distal colon cancer, HRs per 5 kg weight gain were 1.18 (95 % CI 1.01–1.37), 1.22 (95 % CI 1.02–1.45), and 1.40 (95 % CI 1.01–1.95), respectively. Weight gain was not significantly associated with survival in men.

**Conclusions:**

Maintaining a healthy weight may benefit CRC survival, at least in women.

**Electronic supplementary material:**

The online version of this article (doi:10.1186/s12885-016-2445-4) contains supplementary material, which is available to authorized users.

## Background

Excess body weight is an established risk factor for colorectal cancer (CRC) in both men and women, and a positive association between body mass index (BMI) and CRC incidence has been found in numerous studies [1]. The association is stronger in men than in women and stronger for colon than for rectal cancers [1]. Furthermore, the association seems to be stronger for distal than for proximal colon cancers [2]. These observations support that the biological mechanisms operating may vary by sex and colorectal subsite.

Less is known about the influence of excess body weight on CRC survival. However, it is possible that the mechanisms linking excess body weight to development of CRC tumors, related to e.g. insulin, insulin-like growth factors, inflammation, and steroid hormones, also influence tumor progression and thereby survival of the disease [3, 4]. Some studies have evaluated the association between BMI at the time of treatment, i.e. around the time of diagnosis, and survival after colon [5–7] or rectal cancer [8, 9], but the results are difficult to interpret since weight loss is a clinical feature of CRC [10]. Thus, the patients’ weight might be a consequence of the disease itself (‘reverse causation’). Moreover, whether maintaining a healthy weight throughout adulthood is important not only for CRC prevention, but also for CRC survival, is not clear from these studies. Pre-diagnostic BMI is probably a better marker of weight across life-course than BMI at the time of diagnosis.

A recent meta-analysis found that pre-diagnostic obesity (BMI ≥30 kg/m^2^) was significantly associated with poorer survival after CRC [11]. However, this meta-analysis only presented results for men and women combined and for CRC overall. Few of the studies that have evaluated the association between pre-diagnostic BMI and CRC survival have examined whether results differ for men and women [12–17] or between CRC subsites [12–14, 17–21]. Finally, although adult weight gain is related to increased colon cancer risk [22], only one study has examined pre-diagnostic weight change and survival after CRC [17].

We have previously studied BMI and weight change in relation to colon cancer risk in the Norwegian Counties Study [23]. The aim of the present study was to examine sex-specific and subsite-specific associations between BMI and weight change measured prior to diagnosis and survival among incident CRC cases from this cohort.

## Methods

### The Norwegian counties study

The Norwegian Counties Study is a population-based Norwegian cohort study described in detail elsewhere [23, 24]. In short, participants were examined by a team of trained nurses at health screenings up to three times between 1974 and 1988. The attendance rate was >80 % at all three screenings, and 92,234 men and women attended at least one screening. At each screening, the participants’ height was measured to the nearest centimeter and weight to the nearest 0.5 kilo. Information on lifestyle factors such as smoking habits and recreational and occupational physical activity during the last year was collected with a questionnaire.

Using the unique personal identification number assigned to all Norwegian citizens, information on each participant’s education was obtained from records of the censuses in 1970, 1980, 1990, and 2001. The most recent information available was used.

### Case identification

CRC cases were identified through linkage with the Cancer Registry of Norway, i.e. cancers coded as 153 or 154 according to the International Classification of Diseases, Seventh edition (ICD-7). The cases were categorized as colon cancer (ICD-7: 153) or rectal cancer (ICD-7: 154). Furthermore, cancers of the appendix, cecum, ascending or transverse colon (including the hepatic and splenic flexures) (153.0, 153.1, and 153.6) were categorized as proximal colon cancer. Cancers that occurred in the descending colon, sigmoid colon, or rectosigmoid junction (153.2 − 153.4) were categorized as distal colon cancer. We only considered the first cancer diagnosis. Participants diagnosed with multiple malignant tumors at the date of first diagnosis were included as CRC cases if all the cancers occurred in the colorectum. Correspondingly, multiple cancers were included as a cancer of one of the subsites (colon, proximal colon, distal colon, or rectum) if they all occurred in the same subsite. The cases were classified according to stage at diagnosis as localized, regional, or distant. For participants with multiple malignant tumors, stage at diagnosis was defined as the stage of the most advanced.

We identified 2786 cases of CRC among the cohort participants. Of these, 69 were diagnosed with multiple malignant tumors.

### Study sample

For each case, we obtained information on weight, smoking, and physical activity level from the most recent screening. This screening was defined as the case’s exposure screening if 1) no information was missing and 2) the screening took place at least 3 years prior to diagnosis. Otherwise, the most recent preceding screening fulfilling these requirements was defined as the exposure screening. Pre-diagnostic BMI (kg/m^2^) was calculated as weight at the exposure screening divided by height squared, using the mean height from all attended screenings. We excluded 54 cases with missing information on weight, smoking, and physical activity at all three screenings, 35 cases diagnosed before information was collected, and 44 cases diagnosed <3 years after the information was collected. We furthermore excluded 5 cases with no height measurements and 6 cases without information on education. We also excluded 23 cases with BMI <18.5 kg/m^2^ at the exposure screening due to concerns that their weight was influenced by pre-existing CRC or other underlying health problems that may influence survival. Finally, we excluded 97 cases with unknown stage at diagnosis, 2 cases with date of diagnosis equal to date of death, and 4 cases with date of emigration prior to their date of diagnosis.

In total, 2516 CRC cases, 1336 men and 1180 women, diagnosed between 1978 and 2012, were included in analyses of BMI and CRC survival (Additional file 1 : Figure S1). In men, 847 of the cases were colon cancers, of which 443 occurred in the proximal and 369 in the distal colon, and 478 cases were rectal cancers. In women, 808 cases were colon cancers, 493 were proximal colon cancers, 294 were distal colon cancers, and 367 were rectal cancers.

For analyses of weight change, we furthermore excluded 622 cases with no weight measurements prior to the exposure screening, leaving 999 men (637 colon cancers, 339 proximal colon cancers, 270 distal colon cancers, 351 rectal cancers) and 895 women (608 colon cancers, 383 proximal colon cancers, 207 distal colon cancers, 283 rectal cancers) (Additional file 2: Figure S2).

We determined the power of detecting an effect estimate per 5 unit increase in BMI of 1.20 with a significance level of 0.05 in our study sample [25]. We used the standard deviation of BMI among CRC cases, 3.2 kg/m^2^ in men and 4.5 kg/m^2^ in women. In men, the power was 0.87 for CRC, 0.69 for colon cancer, 0.41 for proximal colon cancer, 0.31 for distal colon cancer, and 0.46 for rectal cancer. In women, the power was 0.97, 0.87, 0.68, 0.45, and 0.60 for CRC, colon cancer, proximal colon cancer, distal colon cancer, and rectal cancer, respectively.

The cases were followed from the date of diagnosis until death, emigration, or December 31, 2012. Information on death and emigration was obtained from the Cause of Death Registry and the National Population Registry. CRC-related death was the primary outcome and death from all causes was the secondary outcome. Deaths with cause 153–154 (ICD-8 and ICD-9) or C18–21 (ICD-10) were considered CRC-related deaths.

### Statistical analysis

Pre-diagnostic BMI was categorized according to WHO guidelines: 18.5 − 24.9 kg/m^2^ (normal weight), 25 − 29.9 kg/m^2^ (overweight), and ≥30 kg/m^2^ (obese) [26]. Weight change was defined as weight at the exposure screening minus initial weight, i.e., weight at the first attended screening, and was categorized as ≤−2.0 kg (weight loss), −1.9 − 1.9 kg (weight maintenance), 2.0–7.9 kg (moderate weight gain), and ≥8.0 kg (large weight gain).

We examined BMI and weight change in relation to risk of death from CRC, our primary outcome, and risk of death from all causes. Hazard ratios (HRs) and 95 % confidence intervals (CIs) were estimated with Cox regression. We used a stratified Cox model with stage of disease at diagnosis as the strata (localized, regional, or distant) and time since diagnosis as the time variable. We adjusted for smoking (never, former, current) and level of recreational and occupational physical activity combined (sedentary, moderately active, or active) [23], both measured at the exposure screening, education (primary schooling (≤9 years), secondary education (10–12 years), or university level education (≥ 13 years)), age at diagnosis (continuous) and year of diagnosis (< 1990, 1990–94, 1995–99, 2000–2004, and ≥2005). Analyses of weight change were also adjusted for initial BMI (continuous). Adjustment for time between the exposure screening and diagnosis did not change the results and was not included in the model. To test for trend, BMI/weight change was modeled continuously and evaluated with a Wald test. All analyses with continuous weight change were restricted to subjects who maintained or gained weight because we hypothesized that weight loss may cause a J or U-shaped relation. We furthermore tested whether the associations with CRC-specific mortality differed in the colon and rectum or in the proximal and distal colon by testing the difference in trends with a Wald statistic. To assess whether associations differed with sex, we included interaction terms between sex and BMI (continuous) or between sex and weight gain (continuous). For CRC, we also examined whether the association between weight gain (continuous) and mortality differed with initial BMI (<25 kg/m^2^ and ≥25 kg/m^2^). Tests of interaction were evaluated with a likelihood ratio test and were done for CRC-specific mortality only. Proportional hazards assumptions were evaluated with a test based on the Schoenfeld residuals. The tests did not indicate violations of the assumption. All tests were two sided, and *P* < 0.05 was considered statistically significant. The analyses were performed with SAS version 9.4 (SAS Institute, Cary, NC).

## Results

### Body mass index

Mean age at diagnosis in the BMI study sample (*n* = 2516) was 66.4 years (range 32.0–85.6) in men and 66.8 years (range 36.0–86.4) in women. During a mean follow-up of 5.8 years, 703 men and 543 women died. Of these, 507 men and 432 women died from CRC. Time from BMI measurement to diagnosis ranged from 3.0 to 37.5 years (mean 17.8 years). Mean pre-diagnostic BMI was similar in men and women, 26.0 kg/m^2^ and 25.7 kg/m^2^, respectively. For both men and women, the proportion with a university education was highest among those with normal weight (Table 1). With increasing BMI, the proportion of sedentary subjects increased, the proportion of current smokers decreased, and mean systolic blood pressure increased. Furthermore, age at diagnosis increased and the proportion with distant stage disease decreased with increasing BMI in women.

**Table 1:** Characteristics of CRC cases by pre-diagnostic BMI, *n* = 2516

	MEN			WOMEN		
	BMI (kg/m^2^)			BMI (kg/m^2^)		
	18.5–24.9	25–29.9	≥30	18.5–24.9	25–29.9	≥30
Number of cases	544	662	130	626	366	188
Follow-up (years), mean	6.1	5.4	5.5	6.1	5.9	5.7
University level education (%)	13.8	12.7	8.5	12.0	6.8	7.4
Current smokers (%)^a^	51.1	38.5	35.4	46.0	32.5	22.9
Sedentary (%)^a^	26.5	26.9	36.2	27.5	32.0	35.6
Systolic blood pressure (mmHg)^a^, mean	134	138	145	128	137	145
Exposure screening (%)						
Screening 1	7.2	7.4	1.5	9.6	3.3	8.0
Screening 2	29.6	26.9	30.0	29.9	27.9	22.9
Screening 3	63.2	65.7	68.5	60.5	68.9	69.1
Age at exposure screening (years), mean	47.9	49.1	49.5	47.8	50.3	50.7
Time between exposure screening and diagnosis (years), mean	17.7	18.2	15.6	18.1	17.5	17.6
Age at diagnosis (years), mean	65.6	67.3	65.1	65.8	67.9	68.2
Stage at diagnosis (%)						
Localized	24.3	24.6	22.3	27.0	25.7	26.6
Regional	52.4	48.5	52.3	46.3	50.5	53.7
Distant	23.3	26.9	25.4	26.7	23.8	19.7

^a^ At exposure screening

In men with proximal colon cancer, pre-diagnostic obesity was associated with higher risk of CRC-related death than normal weight, HR = 1.85 (95 % CI 1.08–3.16) (Table 2). BMI was not significantly associated with CRC-specific mortality in men with CRC, colon cancer, distal colon cancer, or rectal cancer. In women, we found no significant associations between BMI and risk of CRC-related death after diagnosis of CRC or cancer of any colon subsite. However, obese women had significantly increased risk of CRC-related death after diagnosis of rectal cancer, HR = 1.93 (95 % CI 1.13–3.30). We found no significant interactions between BMI and sex (P_interaction_ ≥ 0.26). The associations between BMI and risk of CRC-related death did not differ significantly with subsite: colon vs. rectum (P_heterogeneity_ ≥ 0.27), proximal vs. distal colon (P_heterogeneity_ ≥ 0.33). In contrast with the results on CRC-related death, pre-diagnostic obesity was associated with higher all-cause mortality in men with CRC and colon cancer (Additional file 3: Table S1). In women, associations between pre-diagnostic BMI and all-cause mortality were similar to the associations found for CRC-specific mortality.

**Table 2:** Hazard ratios and 95 % confidence intervals for CRC-specific mortality by pre-diagnostic BMI

	BMI (kg/m^2^)				
	18.5–24.9	25–29.9	≥30	Per 5 kg/m^2^	P_trend_^a^
MEN					
CRC, *n* = 1336					
Person-years	3320	3563	710		
Number of CRC-related deaths	198	254	55		
HR (95 % CI)^b^	1 (Ref)	0.98 (0.81, 1.18)	1.01 (0.74, 1.37)	0.99 (0.86, 1.14)	0.87
Colon cancer, *n* = 847					
Person-years	1936	2144	433		
Number of CRC-related deaths	125	158	39		
HR (95 % CI)^b^	1 (Ref)	1.02 (0.80, 1.30)	1.26 (0.87, 1.83)	1.07 (0.90, 1.28)	0.43
Proximal colon cancer, *n* = 443					
Person-years	1072	1058	160		
Number of CRC-related deaths	63	80	19		
HR (95 % CI)^b^	1 (Ref)	1.17 (0.82, 1.66)	1.85 (1.08, 3.16)	1.19 (0.93, 1.54)	0.17
Distal colon cancer, *n* = 369					
Person-years	848	996	272		
Number of CRC-related deaths	53	65	18		
HR (95 % CI)^b^	1 (Ref)	0.91 (0.63, 1.33)	0.91 (0.52, 1.59)	0.98 (0.75, 1.30)	0.91
Rectal cancer, *n* = 478					
Person-years	1374	1371	273		
Number of CRC-related deaths	73	94	16		
HR (95 % CI)^b^	1 (Ref)	0.99 (0.71, 1.37)	0.75 (0.43, 1.33)	0.93 (0.73, 1.17)	0.52
WOMEN					
CRC, *n* = 1180					
Person-years	3793	2175	1064		
Number of CRC-related deaths	220	138	74		
HR (95 % CI)^b^	1 (Ref)	1.07 (0.85, 1.33)	1.31 (0.99, 1.72)	1.04 (0.93, 1.17)	0.51
Colon cancer, *n* = 808					
Person-years	2469	1386	760		
Number of CRC-related deaths	153	90	50		
HR (95 % CI)^b^	1 (Ref)	0.97 (0.74, 1.27)	1.25 (0.89, 1.74)	1.02 (0.89, 1.18)	0.75
Proximal colon cancer, *n* = 493					
Person-years	1279	876	372		
Number of CRC-related deaths	93	53	29		
HR (95 % CI)^b^	1 (Ref)	0.89 (0.63, 1.28)	1.23 (0.80, 1.91)	1.02 (0.84, 1.24)	0.82
Distal colon cancer, *n* = 294					
Person-years	1160	467	369		
Number of CRC-related deaths	54	34	17		
HR (95 % CI)^b^	1 (Ref)	1.33 (0.84, 2.11)	1.18 (0.64, 2.16)	1.06 (0.83, 1.35)	0.65
Rectal cancer, *n* = 367					
Person-years	1271	788	283		
Number of CRC-related deaths	67	47	24		
HR (95 % CI)^b^	1 (Ref)	1.42 (0.95, 2.13)	1.93 (1.13, 3.30)	1.18 (0.96, 1.47)	0.12

^a^ Wald P-value for BMI as continuous variable

^b^ Stratified Cox model (year of diagnosis: < 1990, 1990–1994, 1995–1999, 2000–2004, ≥ 2005). Adjustment for age at diagnosis, stage of disease at diagnosis (localized, regional, or distant), smoking (never, former, or current), physical activity level (sedentary, moderately active, or active), education (≤ 9, 10-12, or ≥ 13 years)

### Weight change

In the weight change study sample (*n* = 1894), 533 men and 414 women died during follow-up. Of these, 372 men and 326 women died from CRC. Mean weight change was 2.5 kg in men and 2.0 kg in women. Cases who lost weight prior to diagnosis had a higher initial BMI and were more likely to be current smokers than cases who maintained or gained weight (Table 3). Furthermore, the highest proportion of sedentary subjects was found among those who gained ≥8 kg. In men, the proportion with distant stage disease was lowest among those who had maintained their weight and the proportion increased with increasing weight gain, whereas in women the proportion with distant stage disease decreased with increasing weight gain.

**Table 3:** Characteristics of CRC cases by pre-diagnostic weight change, *n* = 1894

	MEN				WOMEN			
	Weight change (kg)				Weight change (kg)			
	≤−2	−1.9–1.9	2.0–7.9	≥8.0	≤−2	−1.9–1.9	2.0–7.9	≥8.0
Number of cases	155	295	418	131	194	256	323	122
Follow-up (years), mean	5.4	6.3	5.4	5.3	5.4	6.1	6.3	5.3
Initial BMI (kg/m^2^), mean	26.5	25.1	24.8	25.8	27.3	24.5	24.3	25.3
University level education (%)	7.1	9.2	12.0	8.4	5.2	7.4	10.5	10.7
Current smokers (%)^a^	61.9	47.8	34.0	25.2	46.9	39.5	33.1	34.4
Sedentary (%)^a^	20.0	20.7	26.6	39.7	31.4	21.9	28.5	37.7
Systolic blood pressure (mmHg)^a^, mean	137	137	138	143	133	134	134	141
Exposure screening (%)								
Screening 2	31.6	42.7	31.3	14.5	39.2	44.5	30.7	8.2
Screening 3	68.4	57.3	68.7	85.5	60.8	55.5	69.3	91.8
Age at exposure screening (years), mean	50.7	50.8	50.2	50.7	51.3	50.5	51.2	50.8
Time between exposure screening and diagnosis (years), mean	16.8	17.2	18.0	16.7	18.3	18.1	17.4	16.5
Age at diagnosis (years), mean	67.5	67.9	68.2	67.4	69.6	68.5	68.5	67.3
Stage at diagnosis (%)								
Localized	27.1	26.8	23.9	21.4	27.3	27.0	28.2	27.9
Regional	45.8	51.2	52.2	51.9	45.4	48.4	48.6	50.8
Distant	27.1	22.0	23.9	26.7	27.3	24.6	23.2	21.3

^a^ At exposure screening

Pre-diagnostic weight gain was not significantly related to CRC-specific mortality in men, but weight loss was associated with significantly increased risk of CRC-related death compared to weight maintenance in men with rectal cancer, HR = 1.78 (95 % CI 1.06–3.00) (Table 4). In women, weight gain was significantly associated with increased risk of death from CRC after diagnosis of CRC, HR = 1.18 (95 % CI 1.01–1.37) per 5 kg weight gain, P_trend_ = 0.03. Moreover, in women with colon cancer, moderate and large weight gain were both associated with significantly increased risk of CRC-related death compared to weight maintenance, HRs = 1.54 (95 % CI 1.08–2.20) and 1.64 (95 % CI 1.03–2.63), respectively, P_trend_ = 0.03. For proximal colon cancer, we observed increased risk in women with moderate weight gain, HR = 1.59 (95 % CI 1.01–2.49), but not in women with large weight gain. In women with distal colon cancer, large weight gain was associated with significant, increased risk of CRC-related death compared to weight maintenance, HR = 2.99 (95 % CI 1.27–7.05), P_trend_ = 0.04. Weight change was not associated with CRC-specific mortality in women with rectal cancer. No significant interactions between weight gain and sex were observed (P_interaction_ ≥ 0.18). The associations did not differ significantly with subsite; colon vs. rectum (P_heterogeneity_ ≥ 0.38), proximal vs. distal colon (P_heterogeneity_ ≥ 0.12). We did not observe significant interactions between BMI and weight gain (P_interaction_ ≥ 0.33). The results on all-cause mortality were similar to the results on CRC-specific mortality, except that weight gain was not significantly associated with higher all-cause mortality in women with CRC overall (Additional file 4: Table S2).

**Table 4:** Hazard ratios and 95 % confidence intervals for CRC-specific mortality by pre-diagnostic weight change

	Weight change (kg)					
	≤−2	−1.9–1.9	2.0–7.9	≥8.0	Per 5 kg^a^	P_trend_^b^
MEN						
CRC, *n* = 999						
Person-years	836	1844	2240	694		
Number of CRC-related deaths	67	109	144	52		
HR (95 % CI)^c^	1.27 (0.93, 1.74)	1 (Ref)	0.95 (0.73, 1.23)	0.93 (0.65, 1.32)	1.01 (0.87, 1.19)	0.86
Colon cancer, *n* = 637						
Person-years	505	1043	1454	360		
Number of CRC-related deaths	38	70	92	35		
HR (95 % CI)^c^	0.96 (0.64, 1.44)	1 (Ref)	0.81 (0.59, 1.13)	1.02 (0.66, 1.58)	1.05 (0.86, 1.28)	0.62
Proximal colon cancer, *n* = 339						
Person-years	304	438	833	120		
Number of CRC-related deaths	14	38	52	19		
HR (95 % CI)^c^	0.59 (0.31, 1.12)	1 (Ref)	0.66 (0.42, 1.05)	0.77 (0.40, 1.48)	0.88 (0.63, 1.23)	0.46
Distal colon cancer, *n* = 270						
Person-years	195	576	575	224		
Number of CRC-related deaths	19	28	33	14		
HR (95 % CI)^c^	1.53 (0.80, 2.93)	1 (Ref)	0.95 (0.55, 1.62)	1.50 (0.75, 3.00)	1.25 (0.94, 1.66)	0.13
Rectal cancer, *n* = 351						
Person-years	330	798	737	325		
Number of CRC-related deaths	28	39	51	17		
HR (95 % CI)^c^	1.78 (1.06, 3.00)	1 (Ref)	1.21 (0.78, 1.87)	0.76 (0.40, 1.44)	0.95 (0.74, 1.23)	0.70
WOMEN						
CRC, *n* = 895						
Person-years	1049	1562	2034	646		
Number of CRC-related deaths	72	91	119	44		
HR (95 % CI)^c^	1.14 (0.82, 1.59)	1 (Ref)	1.33 (1.01, 1.76)	1.41 (0.97, 2.05)	1.18 (1.01, 1.37)	0.03
Colon cancer, *n* = 608						
Person-years	670	1124	1270	473		
Number of CRC-related deaths	49	53	85	30		
HR (95 % CI)^c^	1.34 (0.88, 2.02)	1 (Ref)	1.54 (1.08, 2.20)	1.64 (1.03, 2.63)	1.22 (1.02, 1.45)	0.03
Proximal colon cancer, *n* = 383						
Person-years	423	673	645	247		
Number of CRC-related deaths	32	33	50	18		
HR (95 % CI)^c^	1.28 (0.76, 2.16)	1 (Ref)	1.59 (1.01, 2.49)	1.49 (0.81, 2.75)	1.18 (0.95, 1.47)	0.14
Distal colon cancer, *n* = 207						
Person-years	231	449	579	197		
Number of CRC-related deaths	15	18	29	12		
HR (95 % CI)^c^	1.52 (0.71, 3.25)	1 (Ref)	1.34 (0.69, 2.60)	2.99 (1.27, 7.05)	1.40 (1.01, 1.95)	0.04
Rectal cancer, *n* = 283						
Person-years	379	438	721	173		
Number of CRC-related deaths	23	37	34	14		
HR (95 % CI)^c^	0.93 (0.52, 1.64)	1 (Ref)	1.00 (0.60, 1.69)	1.29 (0.67, 2.49)	1.12 (0.82, 1.53)	0.49

^a^ Analyses restricted to cases who maintained or gained weight

^b^ Wald P-value for weight gain as continuous variable

^c^ Stratified Cox model (year of diagnosis: < 1990, 1990–1994, 1995–1999, 2000–2004, ≥2005). Adjustment for age at diagnosis, stage of disease at diagnosis (localized, regional, distant), smoking (never, former, or current), physical activity level (sedentary, moderately active, or active), education (≤9, 10–12, or ≥13 years), and initial BMI (continuous)

## Discussion

In this study, obesity prior to diagnosis was significantly associated with higher CRC-specific mortality in men with proximal cancer and in women with rectal cancer. Furthermore, pre-diagnostic weight gain was significantly associated with higher CRC-specific mortality in women with CRC, colon cancer, proximal colon cancer, or distal colon cancer. Weight gain was not related to CRC-specific mortality in men. However, men with rectal cancer who had lost weight prior to diagnosis had increased risk of CRC-related death.

The association between BMI and CRC risk is stronger in men than in women [1], but whether there are sex differences regarding excess body weight and survival after CRC is not known. Our results did not indicate a stronger association in men. Results from previous studies are inconsistent. Men who were obese prior to diagnosis of CRC have been found to have poorer survival [14], while other studies have found weak or no associations [13, 15, 16, 27]. Likewise, both significantly poorer survival [12, 17, 19] and no associations [14, 16, 28] have been found in women who were obese prior to CRC diagnosis. Differences between the cases in these studies in terms of e.g. age at diagnosis, stage of disease, treatment, and subsite distribution may explain the disparities in the results. Another possible explanation is differences in the timing of BMI measurement in relation to diagnosis which may also influence the results.

We expected to find stronger associations between BMI and survival for colon cancer than for rectal cancer. However, in women, the opposite was observed. Results from previous studies are inconsistent. Pre-diagnostic obesity has been found to be associated with significantly poorer survival after both colon [14, 17, 19–21] and rectal cancer [12–14, 17], but no association with survival after colon cancer [13, 29] or after rectal cancer [19, 21] has also been reported. Only two studies have presented results on pre-diagnostic BMI in relation to survival after cancer in subsites of the colon [14, 18]. These studies found that BMI was significantly associated with poorer survival after distal colon cancer, whereas a non-significant association was found for proximal colon cancer [14, 18]. Subsite-differences in the association between BMI and survival after diagnosis of CRC may be related to molecular features of CRC tumors. Microsatellite instable tumors tend to be proximal and are associated with better survival than microsatellite stable tumors [30]. Moreover, BMI has been found to be associated with an increased risk of microsatellite stable tumors, but not microsatellite instable tumors [31, 32].

We found that weight gain before diagnosis of CRC was related to poorer survival in women, but not in men. Only one study has previously evaluated pre-diagnostic weight change in relation to survival after CRC [17]. In this study, weight gain since age 20 years was not associated with poorer survival in either men or women. Weight gain in adulthood is mainly an accumulation of fat mass. The distribution of the accumulated fat may influence CRC survival, since various fat depots have different metabolic characteristics [33]. We speculate that weight gain may be more strongly related to harmful fat distribution in women than in men. Also, the timing of weight gain could be important. Possibly, weight gain during menopause is especially harmful. Menopause is associated with an increase in visceral adipose tissue [34], which is particularly related to metabolic disturbances [33]. Many of the women in our study sample likely became postmenopausal between weight measurements; median age at the last weight measurement was 51.7 years for women.

Excess body weight may be linked to increased cancer risk through changes in steroid hormones, insulin, insulin-like growth factors, leptin and adiponectin, or pro-inflammatory cytokines [35]. It is possible that such changes also influence prognosis and survival of cancer, but the biological role of excess body weight on cancer survival is under debate. Also, there may have been differences between normal weight and obese cases in terms comorbidities, receipt of optimal treatment and complications as a result of treatment. Whether this could explain the associations between obesity and mortality among men with proximal colon cancer and women with rectal cancer could not be explored in our study since we did not have information on treatment.

It is of course possible that excess body weight does not influence survival. Weight gain was associated with survival across CRC subsites in women, but we did not observe strong or consistent associations in men. The significant associations we observed may have been due to chance. We performed a large number of statistical tests and would expect to find significant associations even if BMI and weight change are not related to CRC survival. However, it is also likely that the causal effect of obesity and weight change may have been underestimated in our study due to collider-stratification bias [36]. The study population was selected based on occurrence of an event, CRC. Suppose CRC is caused by both obesity and an uncontrolled risk factor. Then CRC is a collider, and conditioning on CRC may result in bias if the uncontrolled risk factor is also a cause of death [36]. The prevalence of the risk factor may be inversely associated with obesity among CRC cases, even when obesity and the risk factor are unrelated in the general population. As a consequence, the association between obesity and risk of death among CRC cases will be biased towards the null or even reversed [37]. Even though we controlled for several important risk factors for CRC and death in our analyses, residual collider-stratification bias cannot be ruled out. However, it is possible that the magnitude of the bias differs with sex or CRC subsite. Future studies should attempt to minimize collider-stratification-bias and must include detailed information on a large number of common causes of CRC and death.

Our study was based on CRC cases from a large cohort study with a very high attendance rate (80 %). Through linkage with the Cancer Registry of Norway we have identified all cases within the cohort. Because of the prospective design, inclusion of cases did not depend upon duration of survival. Thus, we consider selection bias to be negligible in our study. We cannot exclude the possibility of reverse causation in our study, but given the high median length of time between measurement and diagnosis, weight at the time of measurement is unlikely to have been influenced by pre-existing disease for the majority of the cases. Consequently, reverse causation is presumably not a concern in our study. Another important strength is the accuracy of the exposures. Weight and height were measured by trained nurses following a strict protocol. Furthermore, information on weight was collected up to three times, thus we could study the effect of pre-diagnostic weight change on CRC survival. We could also examine sex differences, as our study included both men and women.

One limitation of our study is the long time between exposure measurement and diagnosis. Thus, changes in BMI over time may have occurred. However, measurements of BMI made earlier in life have been found to be strongly related to measurements later in life [38, 39]. Moreover, additional adjustment for time between measurement and diagnosis did not change our results. If fat distribution is important in relation to survival after CRC, we may have underestimated the effect of excess body weight, because BMI does not capture fat distribution. We could not explore whether measures of abdominal adiposity like waist circumference or waist-to-hip ratio are more important prognostic factors than BMI, since we did not have information on anthropometry beside BMI. Another limitation is that we could not assess the effect of treatment, a possible confounder, since we did not have information on type of treatment the cases received. Finally, we did not have detailed information on the participants’ diet and could therefore not adjust for important risk factors for CRC like intake of red meat and alcoholic drinks.

## Conclusions

In contrast with what is known about excess body weight and risk of CRC, we found little evidence of poorer survival among men who were obese or gained weight prior to diagnosis of CRC in this study. However, pre-diagnostic weight gain was related to poorer survival after CRC in women. Thus, our study supports that having maintained a healthy weight throughout adulthood may be beneficial for CRC survival, at least for women. The reason for the lack of observed effect in men in our study is not clear. Potential sex differences must be further investigated. Future studies should include detailed information on treatment and a large number of common causes of CRC and death.

## Abbreviations

BMI, body mass index; CI, confidence interval; CRC, colorectal cancer; HR, hazard ratio; ICD, International Classification of Diseases

## Additional files

Additional file 1: Figure S1. Flow chart illustrating number of cases in each colorectal cancer subsite for the BMI study sample. (PDF 38 kb) Additional file 2: Figure S2. Flow chart illustrating number of cases in each colorectal cancer subsite for the weight change study sample. (PDF 36 kb) Additional file 3: Table S1. Hazard ratios and 95% confidence intervals for all-cause mortality by BMI. (PDF 101 kb) Additional file 4: Table S2 Hazard ratios and 95% confidence intervals for all-cause mortality by weight change. (PDF 104 kb)
