# Supplementary material for: Pre-diagnostic body mass index and weight change in relation to colorectal cancer survival among incident cases from a population-based cohort study
Source: BMC Cancer. 2016 Jul 7;16:402. doi: 10.1186/s12885-016-2445-4 (PMC4936308; doi:10.1186/s12885-016-2445-4)
Supplement: Additional file 2: Figure S2. — Flow chart illustrating number of cases in each colorectal cancer subsite for the weight change study sample. (PDF 36 kb) [file 12885_2016_2445_MOESM2_ESM.pdf]

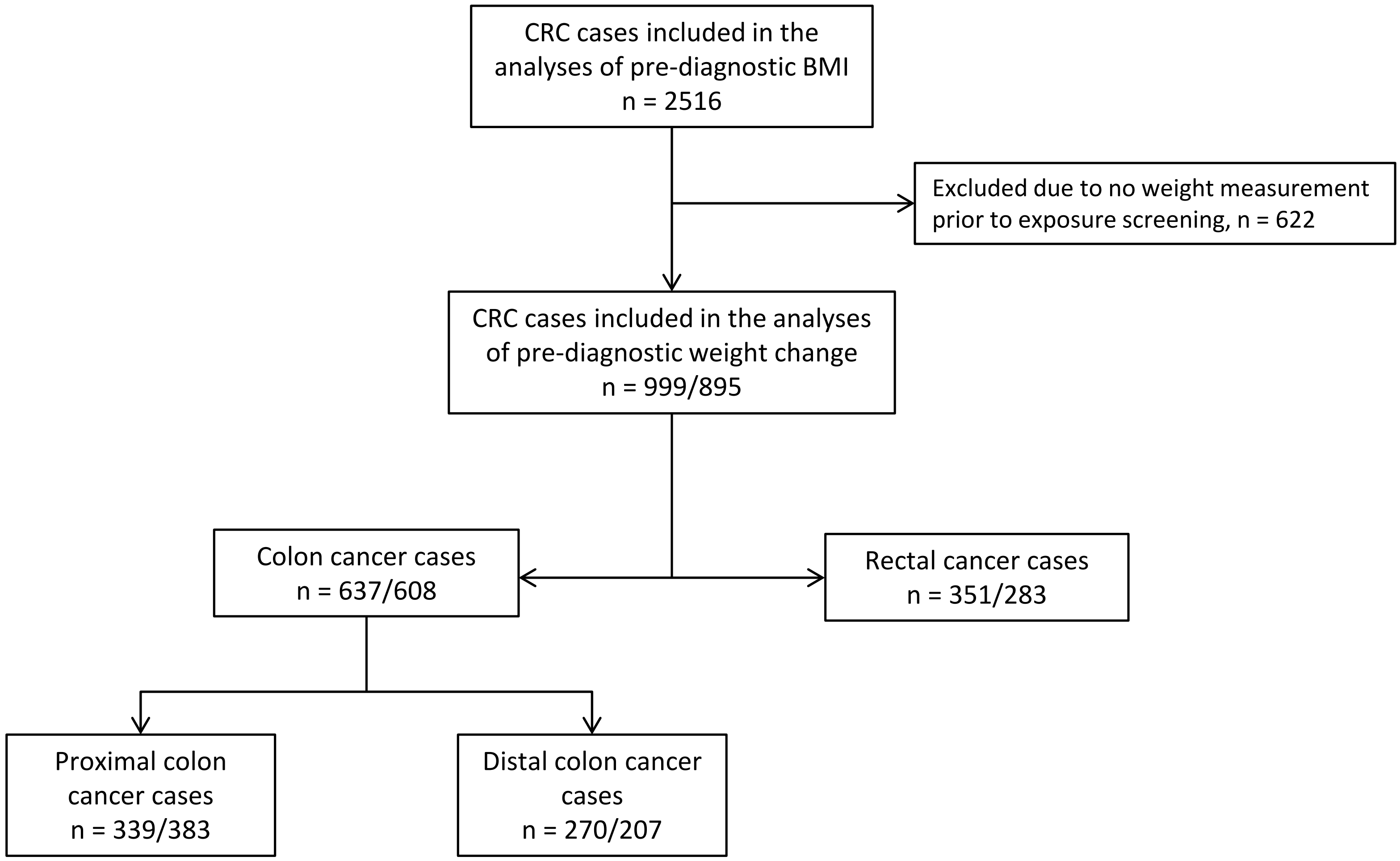

Figure S2: Flow chart illustrating number of cases in each colorectal cancer (CRC) subsite, weight change study sample (n = men/women)
