# Supplementary material for: Pre-diagnostic body mass index and weight change in relation to colorectal cancer survival among incident cases from a population-based cohort study
Source: BMC Cancer. 2016 Jul 7;16:402. doi: 10.1186/s12885-016-2445-4 (PMC4936308; doi:10.1186/s12885-016-2445-4)
Supplement: Additional file 3: Table S1. — Hazard ratios and 95% confidence intervals for all-cause mortality by BMI. (PDF 101 kb) [file 12885_2016_2445_MOESM3_ESM.pdf]

Table S1: Hazard ratios and 95% confidence intervals for all-cause mortality by BMI

	BMI (kg/m^2)				
	**18.5–24.9**	**25–29.9**	**≥ 30**	**Per 5 kg/m^2**	**P_{trend}***
**MEN**					
**CRC, n = 1336**					
Person-years	3320	3563	710		
Number of all-cause deaths	269	348	86		
HR (95% CI)†	1 (Ref)	1.01 (0.86, 1.19)	1.29 (1.00, 1.66)	1.11 (0.99, 1.24)	0.09
**Colon cancer, n = 847**					
Person-years	1936	2144	433		
Number of all-cause deaths	176	212	58		
HR (95% CI)†	1 (Ref)	0.98 (0.79, 1.20)	1.44 (1.06, 1.96)	1.15 (0.99, 1.33)	0.07
**Proximal colon cancer, n = 443**					
Person-years	1072	1058	160		
Number of all-cause deaths	92	103	26		
HR (95% CI)†	1 (Ref)	0.99 (0.73, 1.34)	1.77 (1.12, 2.81)	1.21 (0.97, 1.50)	0.09
**Distal colon cancer, n = 369**					
Person-years	848	996	272		
Number of all-cause deaths	73	94	30		
HR (95% CI)†	1 (Ref)	1.01 (0.73, 1.38)	1.27 (0.81, 1.99)	1.15 (0.93, 1.44)	0.20
**Rectal cancer, n = 478**					
Person-years	1374	1371	273		
Number of all-cause deaths	92	134	26		
HR (95% CI)†	1 (Ref)	1.17 (0.88, 1.54)	1.10 (0.69, 1.74)	1.08 (0.89, 1.31)	0.43
**WOMEN**					
**CRC, n= 1180**					
Person-years	3793	2175	1064		
Number of all-cause deaths	281	172	90		
HR (95% CI)†	1 (Ref)	1.05 (0.86, 1.29)	1.21 (0.95, 1.56)	1.04 (0.94, 1.15)	0.49
**Colon cancer, n = 808**					
Person-years	2469	1386	760		
Number of all-cause deaths	188	113	58		
HR (95% CI)†	1 (Ref)	1.01 (0.79, 1.29)	1.15 (0.85, 1.57)	1.03 (0.90, 1.17)	0.69
**Proximal colon cancer, n = 493**					
Person-years	1279	876	372		
Number of all-cause deaths	115	71	34		
HR (95% CI)†	1 (Ref)	0.97 (0.71, 1.33)	1.21 (0.81, 1.80)	1.05 (0.90, 1.24)	0.52

(Continued on following page)

Supplementary table 1: Hazard ratios and 95% confidence intervals for all-cause mortality by BMI (continued)

	BMI (kg/m^2)				
	**18.5–24.9**	**25–29.9**	**≥ 30**	**Per 5 kg/m^2**	**P_{trend}***
**Distal colon cancer, n = 294**					
Person-years	1160	467	369		
Number of all-cause deaths	67	39	20		
HR (95% CI)†	1 (Ref)	1.23 (0.81, 1.87)	1.08 (0.62, 1.86)	1.02 (0.82, 1.28)	0.83
**Rectal cancer, n = 367**					
Person-years	1271	788	283		
Number of all-cause deaths	93	58	31		
HR (95% CI)†	1 (Ref)	1.24 (0.87, 1.78)	1.62 (1.01, 2.60)	1.13 (0.93, 1.37)	0.21

* Wald P-value for BMI as continuous variable.

† Stratified Cox model (stage: localized, regional, or distant). Adjustment for age at diagnosis, year of diagnosis (< 1990, 1990–1994, 1995–1999, 2000–2004, ≥ 2005), smoking (never, former, or current), physical activity level (sedentary, moderately active, or active), education (≤ 9, 10-12, or ≥ 13 years).
