# Supplementary material for: Pre-diagnostic body mass index and weight change in relation to colorectal cancer survival among incident cases from a population-based cohort study
Source: BMC Cancer. 2016 Jul 7;16:402. doi: 10.1186/s12885-016-2445-4 (PMC4936308; doi:10.1186/s12885-016-2445-4)
Supplement: Additional file 4: Table S2 — Hazard ratios and 95% confidence intervals for all-cause mortality by weight change. (PDF 104 kb) [file 12885_2016_2445_MOESM4_ESM.pdf]

Table S2: Hazard ratios and 95% confidence intervals for all-cause mortality by weight change

	Weight change (kg)					
	**≤ -2**	**-1.9–1.9**	**2.0–7.9**	**≥ 8.0**	**Per 5 kg**	**P_{trend}**
**MEN**						
**CRC, n = 999**						
Person-years	836	1844	2240	694		
Number of all-cause deaths	97	158	204	74		
HR (95% CI)‡	1.20 (0.93, 1.56)	1 (Ref)	0.99 (0.80, 1.23)	0.99 (0.74, 1.33)	1.05 (0.92, 1.19)	0.46
**Colon cancer, n = 637**						
Person-years	505	1043	1454	360		
Number of all-cause deaths	58	103	129	46		
HR (95% CI)‡	0.98 (0.70, 1.37)	1 (Ref)	0.83 (0.63, 1.09)	0.99 (0.69, 1.44)	1.03 (0.86, 1.22)	0.76
**Proximal colon cancer, n = 339**						
Person-years	304	438	833	120		
Number of all-cause deaths	25	53	68	26		
HR (95% CI)‡	0.64 (0.38, 1.06)	1 (Ref)	0.65 (0.44, 0.95)	0.92 (0.53, 1.59)	0.90 (0.68, 1.19)	0.47
**Distal colon cancer, n = 270**						
Person-years	195	576	575	224		
Number of all-cause deaths	27	44	53	18		
HR (95% CI)‡	1.38 (0.81, 2.34)	1 (Ref)	1.02 (0.66, 1.55)	1.09 (0.61, 1.95)	1.15 (0.89, 1.49)	0.27
**Rectal cancer, n = 351**						
Person-years	330	798	737	325		
Number of all-cause deaths	38	55	72	27		
HR (95% CI)‡	1.62 (1.04, 2.51)	1 (Ref)	1.32 (0.91, 1.91)	0.93 (0.56, 1.55)	1.03 (0.85, 1.26)	0.76
**WOMEN**						
**CRC, n = 895**						
Person-years	1049	1562	2034	646		
Number of all-cause deaths	98	121	147	48		
HR (95% CI)‡	1.12 (0.84, 1.48)	1 (Ref)	1.17 (0.91, 1.49)	1.16 (0.82, 1.64)	1.10 (0.96, 1.26)	0.17
**Colon cancer, n = 608**						
Person-years	670	1124	1270	473		
Number of all-cause deaths	68	69	99	34		
HR (95% CI)‡	1.44 (1.01, 2.05)	1 (Ref)	1.37 (1.00, 1.88)	1.45 (0.95, 2.23)	1.19 (1.02, 1.39)	0.03
**Proximal colon cancer, n = 383**						
Person-years	423	673	645	247		
Number of all-cause deaths	46	46	58	22		
HR (95% CI)‡	1.27 (0.82, 1.97)	1 (Ref)	1.26 (0.85, 1.87)	1.44 (0.85, 2.45)	1.18 (0.98, 1.43)	0.09

(Continued on following page)

Supplementary table 2: Hazard ratios and 95% confidence intervals for all-cause mortality by weight change (continued)

	Weight change (kg)					
	**≤ -2**	**-1.9–1.9**	**2.0–7.9**	**≥ 8.0**	**Per 5 kg***	**P_{trend}†**
**Distal colon cancer, n = 207**						
Person-years	231	449	579	197		
Number of all-cause deaths	20	21	35	12		
HR (95% CI)‡	1.97 (1.00, 3.88)	1 (Ref)	1.62 (0.88, 2.97)	2.53 (1.13, 5.68)	1.34 (0.99, 1.82)	0.06
**Rectal cancer, n = 283**						
Person-years	379	438	721	173		
Number of all-cause deaths	30	51	47	14		
HR (95% CI)‡	0.74 (0.45, 1.21)	1 (Ref)	0.91 (0.58, 1.42)	0.96 (0.51, 1.79)	0.95 (0.70, 1.28)	0.72

* Analyses restricted to cases who maintained or gained weight.

† Wald P-value for weight gain as continuous variable.

‡ Stratified Cox model (stage: localized, regional, or distant). Adjustment for age at diagnosis, year of diagnosis (<1990, 1990-1994, 1995–1999, 2000–2004, ≥ 2005), smoking (never, former, or current), physical activity level (sedentary, moderately active, or active), education (≤ 9, 10-12, or ≥ 13 years), and initial BMI (continuous).
